# Preoperative NarxCare overdose risk scores greater than 100 are associated with worse PROMs improvements and dissatisfaction after primary THA

**DOI:** 10.1007/s00402-026-06372-7

**Published:** 2026-06-18

**Authors:** Shujaa T. Khan, Ahmed K. Emara, Shlok V. Patel, Khaled A. Elmenawi, Alvaro Ibaseta, Ignacio Pasqualini, Chao Zhang, Nicolas S. Piuzzi

**Affiliations:** Department of Orthopaedic Surgery, Cleveland Clinic, Cleveland, USA

**Keywords:** Narx, opioid, hip, replacement, arthroplasty, PROM, satisfaction

## Abstract

**Introduction:**

The NarxCare Overdose Risk Score (ORS) is a weighted measure of patient-specific prescription drug use. In patients undergoing primary total hip arthroplasty (THA), this study aimed to evaluate (1) the trend of the ORS from preoperative to 1-year postoperatively, and (2) the association of preoperative ORS with achievement of clinically meaningful improvements in patient reported outcome measures (PROMs), and satisfaction at 1-year.

**Methods:**

All patients who underwent primary unilateral elective THA at a USA tertiary healthcare system from January 2016-December 2022 were eligible. Patients with incomplete PROMs or missing baseline NarxCare ORS were excluded, leading to the inclusion of 5,424 patients. Multivariable regression models were used to assess the association between ORS and 1-year PROMs, including achievement of the minimum clinically important difference (MCID) and Patient Acceptable Symptom State (PASS) for the Hip Disability and Osteoarthritis Outcome Score (HOOS) subdomain scores, as well as satisfaction.

**Results:**

Preoperatively, 46.1% (*n* = 2501) had a NarxCare ORS of 0, 24.6% (*n* = 1336) had scores between 100 and 199, and 16.2% (*n* = 876) in the 200–299 range. After adjusting for confounding variables, a preoperative ORS of 100–199 was associated with failure to achieve MCID in HOOS Pain (OR 1.91;CI 1.32–2.77;*p* = 0.001), and HOOS JR (OR 1.54;1.10–2.15;*p* = 0.012). Preoperative ORS of 100–199 was also associated with failure to reach PASS threshold in HOOS Pain (OR 1.45;1.23–1.71;*p* < 0.001), PS (OR 1.47;1.22–1.75;*p* < 0.001) and JR (OR 1.26;1.08–1.49;*p* = 0.004), with the odds of failure continually increasing as the ORS increases. Patients with a preoperative ORS of 100–199 were 58% more likely to be dissatisfied at 1-year (OR 1.58;1.26–1.99;*p* < 0.001) compared to prescription drug use naïve patients.

**Conclusions:**

Increasing preoperative NarxCare ORS, a measure of prescription drug use, is inversely associated with patient perceived improvement after THA. An ORS of just 100 may significantly decrease the chances of clinically meaningful improvements in hip pain and function, as well as satisfaction at 1-year. A multidisciplinary approach is warranted to mitigate the detrimental effects of sedatives, opioids, or stimulant drug use and surgeons may use this easily accessible score to guide a patient centered discussion regarding postoperative improvements.

**Level of evidence:**

III.

**Supplementary Information:**

The online version contains supplementary material available at 10.1007/s00402-026-06372-7.

## Introduction

Prescription drug use within the United States (US) is widely common, with roughly 16 million Americans over the age of 12 abusing prescriptions in a given year [1]. It is suggested that up to 2 in 5 surgical candidates in the preoperative period may present with unhealthy use of substances [2]. Specifically in orthopedics, opioid medications are frequently prescribed for managing pain, however, their use is closely linked to the ongoing opioid epidemic, which has spurred a shift in medical and legislative efforts to curtail opioid-related morbidity and mortality [3, 4].

Preoperative opioid use has been linked to greater risks for thromboembolic complications, prolonged length of stay, higher readmission rates, increased revision rates, and higher costs of care in the 90-day THA postoperative period [5, 6]. However, similar associations with nonopioid prescription-drugs remain under-studied and prescription drug use is not a dichotomous variable but rather a multifaceted issue influenced by dose, type, duration, and patterns of drug use [7]. The NarxCare Overdose Risk Score (ORS) is a weighted scalar measure of patient-specific prescription drug use, calculated using a complex algorithm based on the number of prescribing providers, dispensing pharmacies, milligram equivalence doses, coprescribed potentiating drugs, and overlapping prescription days of narcotics, sedatives and stimulants [8, 9]. This readily available score in electronic medical records offers a more nuanced understanding of prescription drug use compared to previous studies which have dichotomized patients into those who use prescription drugs and those who do not [10, 11]. Therefore, by not disregarding several attributes within the group of patients who use those drugs, the NarxCare ORS allows a holistic understanding of the potential impact of sedatives, stimulants and narcotics on THA outcomes.

Although a recent study demonstrated that a NarxCare ORS > 300 was associated with increased healthcare utilization following THA, including higher readmission rates and prolonged hospital stays, the impact of NarxCare ORS on patient-reported outcome measures (PROMs) has not been explored [12]. Understanding this relationship is crucial, as PROMs are increasingly used to gauge the success of arthroplasty from the patient’s perspective. In the current landscape of value-based healthcare, understanding the factors that directly influence postoperative outcomes and the success of THA is increasingly vital for developing optimal practices and delivering high-quality, cost-effective care [13]. This study aims to address these gaps by evaluating: (1) the trend of the NarxCare ORS from preoperative to 1-year postoperatively, and (2) the association between preoperative ORS and the achievement of clinically meaningful improvements in PROMs and patient satisfaction at 1 year.

## Methods

### Study design and setting

All individuals who underwent a primary THA at an American tertiary academic center from January 2016 through December 2022 were retrospectively reviewed. The study cohort was obtained using a validated institutional prospective data-collection system (Orthopaedic Minimal Data Set Episode of Care [OME]) that has been previously described and validated [14, 15]. The OME records patient demographic characteristics, baseline comorbidities, in-hospital metrics (i.e., surgical details, LOS, and discharge disposition), readmission, and ED visits up to 90 days postoperatively, as well as PROMs up to 1 year postoperatively. Institutional review board approval was obtained.

### Study population

**Fig. 1:**
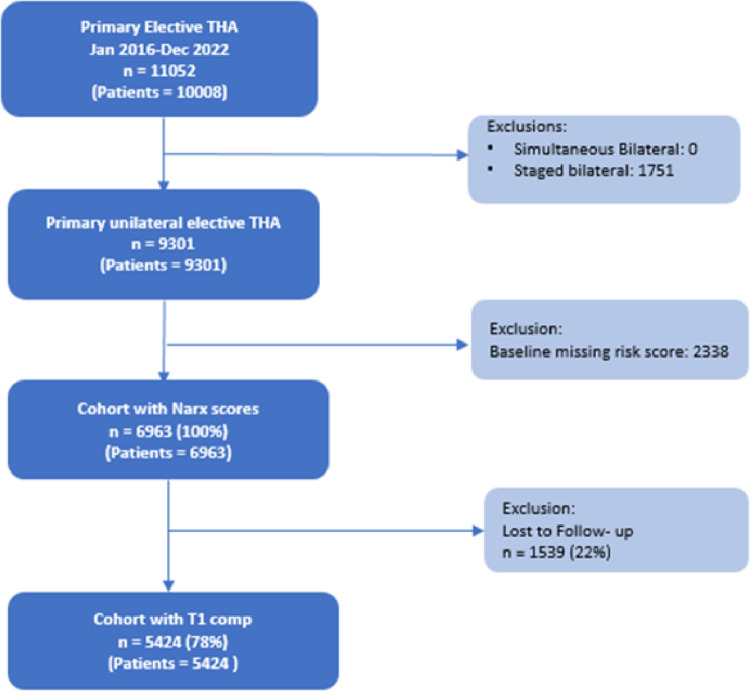
Strengthening the reporting of observational studies in epidemiology (STROBE) diagram

All patients who underwent primary THA were initially eligible for inclusion (*n* = 10008). Patients who underwent simultaneous bilateral or staged bilateral THA (*n* = 1751), missing baseline NarxCare ORS (*n* = 2338), or were lost to follow-up (*n* = 1539) were excluded, leaving 5424 for inclusion in the final analysis (Fig. 1).

### Variables

Demographic characteristics recorded were age, sex, BMI, race, education, smoking status, area deprivation index (ADI), Charlson Comorbidity Index (CCI), insurance. Additionally, diagnosis, anesthesia type, and PROM phenotypes were also recorded. ADI is a measure of socioeconomic deprivation with larger scores indicating greater deprivation in the neighborhood [16]. Patients can be grouped into 8 PROM phenotypes representing above (+) or below (−) the median score for the different PROMs subscales for that specific cohort [17] (Appendix Table 1).

### NarxCare overdose risk score

Scores were obtained from electronic medical records at the time of the initial surgical admission and patients were then stratified into seven preoperative NCS categories: 0 (patients who were naïve to prescription drugs), 1 to 99, 100 to 199, 200 to 299, 300 to 399, 400 to 499, and ≥ 500 based on previous literature [12, 18]. These scores were generated using the NarxCare platform (Bamboo Health), which queries the prescription drug monitoring program (PDMP) during each patient encounter. The scores range from 0 to 999, where 0 represents individuals with no history of prescription drug use, and higher scores signify an increased risk of prescription drug overdose. The algorithm behind these scores evaluates both current and historical prescription drug use (including opioids, sedatives, and stimulants) recorded in the PDMP, factoring in dosage (measured in milligram equivalencies), overlapping prescriptions, and the number of prescribing providers and dispensing pharmacies [4, 8, 9, 12]. For clinical context, Bamboo Health’s sample high-ORS profiles include examples such as an ORS of 710 associated with > 6 dispensations, 60 days of benzodiazepine–narcotic overlap, 3 dispensing pharmacies, 240 total days’ supply of short-acting drugs, and 9 narcotic/sedative prescription combinations. Another sample profile included > 4 opioid or sedative dispensing pharmacies in a 90-day period, > 5 opioid or sedative providers in a year, and > 100 total morphine milligram equivalents with a 40 MME/day average. These examples are provided for clinical context only, as the ORS is generated using a weighted PDMP-based model and should be interpreted alongside the patient’s full clinical history [9].

### Outcomes of interest

Primary outcomes were achievement of Minimum Clinically Important Difference (MCID) and Patient Acceptable Symptom State (PASS) thresholds in a set of PROMs at 1 year. These PROMs included Hip Injury and Osteoarthritis Outcome Score (HOOS) Pain, HOOS Physical Function Short form (HOOS-PS), and HOOS for Joint Replacement (HOOS-JR). Secondary outcome was satisfaction at 1-year [19, 20] (Appendix Table 2).

The HOOS is scored 0-100 with 0 indicating the worst symptoms and 100 indicating no symptoms. It has proven validity and responsiveness and is widely regarded as a valuable tool to evaluate outcomes after THA [21].

Clinical importance was determined using clinical thresholds, MCID – the smallest change in outcome perceived as beneficial by the patient, and PASS threshold – the highest level of symptoms beyond which a patient considers themselves well. To identify PASS, we asked the patients “taking into account all the activity you have during your daily life, your level of pain and also your activity limitations and participation restrictions, do you consider the current state of your hip satisfactory?”, which could only be answered with yes or no. MCID threshold for improvements in HOOS Pain (1y minus baseline) were 8.35, 9.47 for HOOS PS delta, and 7.76 for HOOS JR delta. The PASS thresholds determined from the satisfaction question were ≥ 80.6 for 1-year HOOS Pain, ≥ 83.6 for 1-year HOOS PS, and ≥ 76.8 for 1-year HOOS JR [22].

### Statistical analysis

Continuous variables were described using medians and interquartile ranges, while categorical variables were reported as counts and percentages. Multivariable regression models were employed to evaluate the association between baseline NarxCare ORS and 1-year PROMs. The models were adjusted for predefined demographic and surgical confounding factors. All statistical analyses were two-sided with a Type I error rate of 0.05.

## Results

### Distribution of baseline NarxCare ORS and trend through 1-year postoperatively

**Fig. 2:**
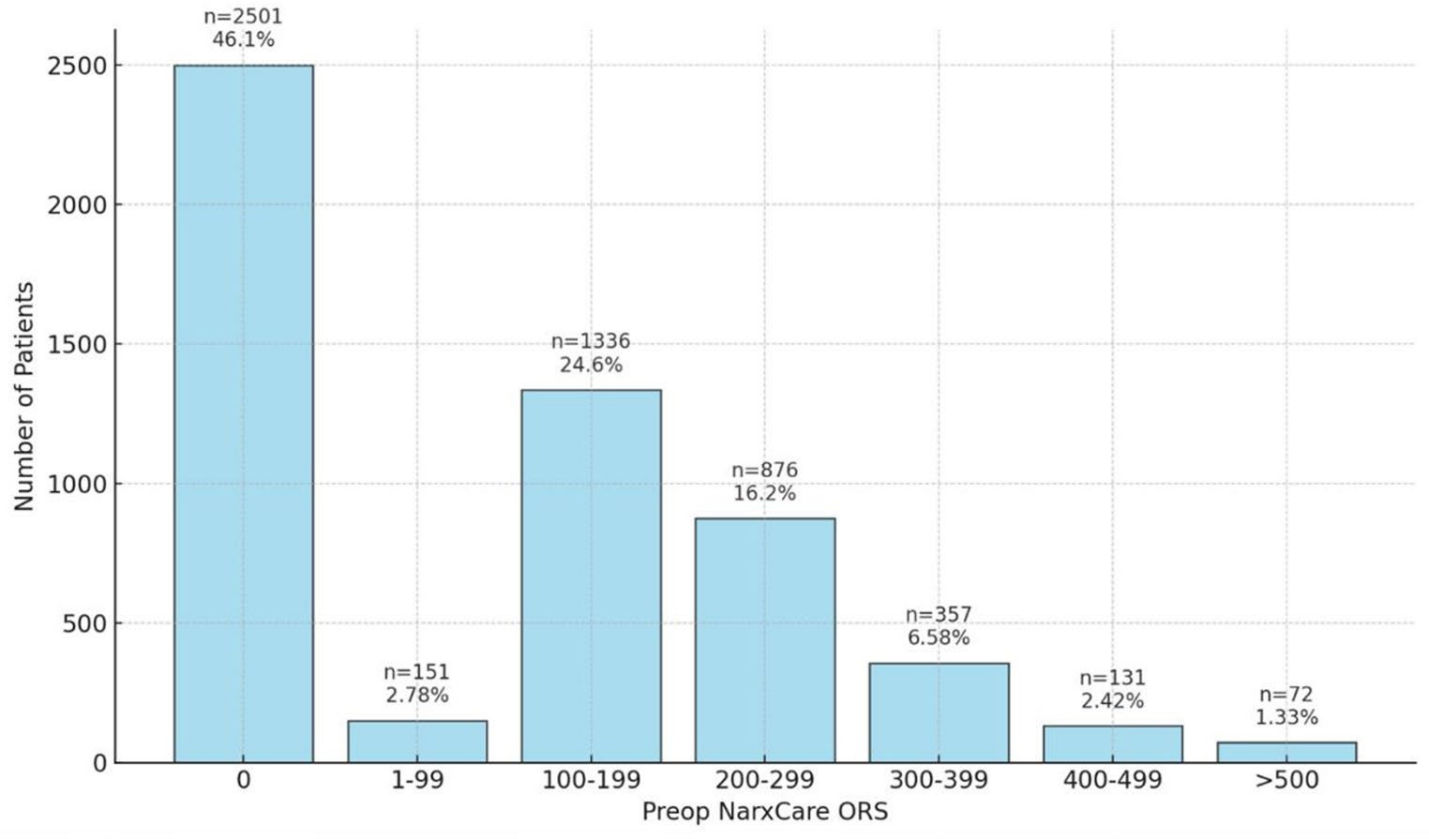
Preoperative distribution of NarxCare overdose risk scores (ORS)

Nearly half of the patients (46.1%, *n* = 2,501) had an ORS of 0, while a substantial proportion (24.6%, *n* = 1,336) had scores between 100 and 199, followed by 16.2% (*n* = 876) with scores in the 200–299 range. Smaller percentages were observed in higher risk categories, with 6.6% (*n* = 357) having scores between 300 and 399, 2.4% (*n* = 131) between 400 and 499, and only 1.3% (*n* = 72) exceeding 500 (Fig. 2).

**Fig. 3:**
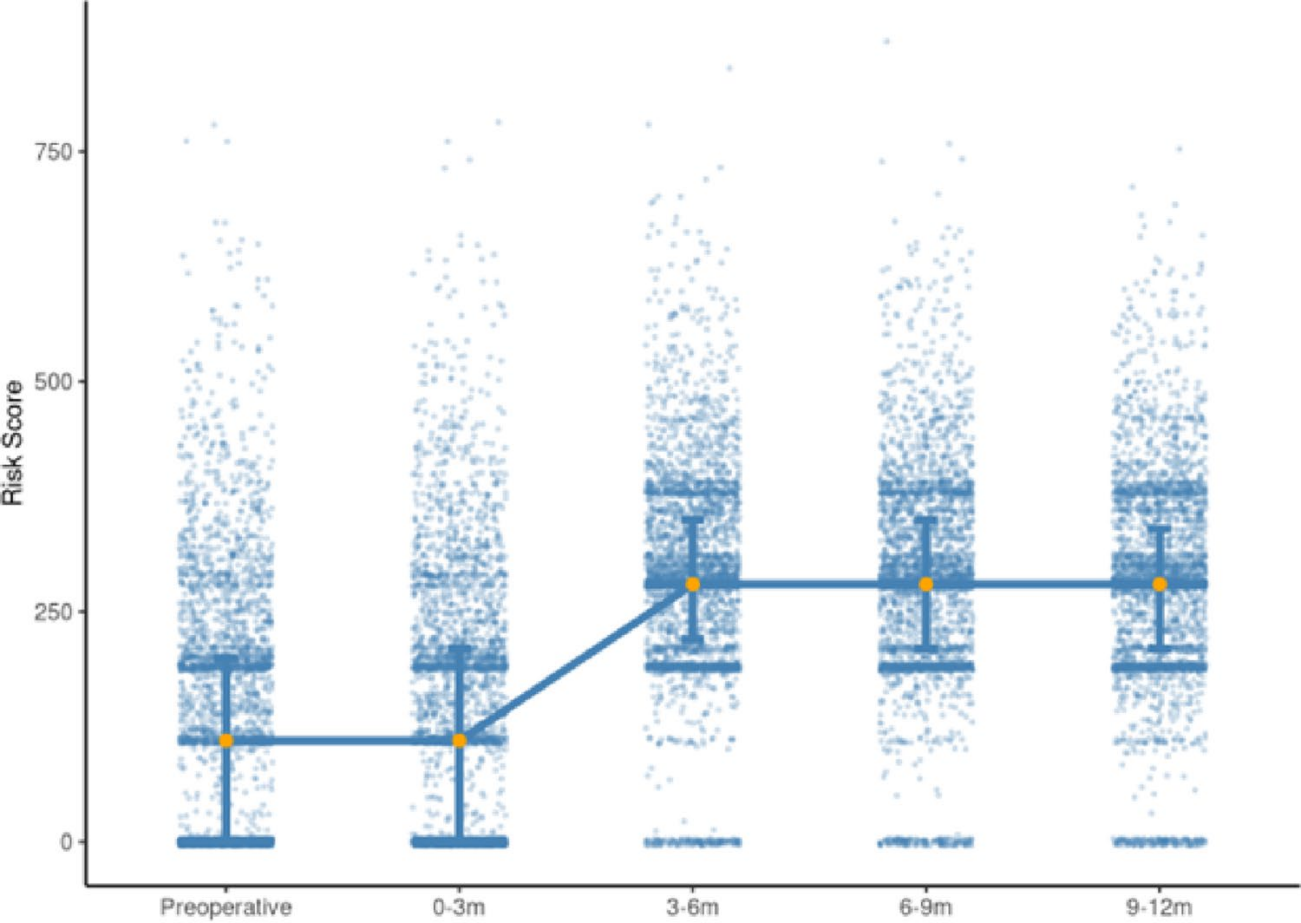
Trend of NarxCare overdose risk scores (ORS) from preoperative to 1-year postoperatively

The ORS starts at median of 110 IQR [0.00;200] preoperatively which increases to its highest of 280 [220;350] at 3–6 months and stays there till 12 months (Fig. 3).

Higher preoperative ORS was associated with worse baseline PROMs. Median baseline HOOS Pain decreased from 40.0 [30.0–50.0] among patients with ORS 0 to 22.5 [7.5–32.5] among patients with ORS > 500. Similar trends were observed for HOOS-PS, which decreased from 53.9 [44.1–66.1] to 38.4 [17.6–53.9], and HOOS-JR, which decreased from 46.7 [36.4–56.0] to 34.5 [19.5–43.3] across the same ORS categories (all *p* < 0.001).

### Baseline NarxCare ORS and achievement of MCID

After adjusting for confounding variables, baseline NarxCare ORS demonstrated varying levels of association with failure to achieve MCID for HOOS Pain, PS, and JR. For pain MCID failure, baseline ORS scores between 100 and 199 were significantly associated with higher odds (OR: 1.91, 95% CI: 1.32–2.77, *p* = 0.001), as were scores ≥ 500 (OR: 4.33, 95% CI: 1.70–11.03, *p* = 0.002). For PS MCID failure, only ORS scores of 400–499 (OR: 2.17, 95% CI: 1.16–4.05, *p* = 0.016) and ≥ 500 (OR: 5.23, 95% CI: 2.71–10.07, *p* < 0.001) showed significant associations. Regarding JR MCID failure, scores across multiple strata were significantly associated: 100–199 (OR: 1.54, 95% CI: 1.10–2.15, *p* = 0.012), 200–299 (OR: 1.56, 95% CI: 1.06–2.30, *p* = 0.024), 300–399 (OR: 1.80, 95% CI: 1.06–3.06, *p* = 0.029), 400–499 (OR: 4.36, 95% CI: 2.22–8.56, *p* < 0.001), and ≥ 500 (OR: 3.71, 95% CI: 1.61–8.57, *p* = 0.002) (Table 1).

### Baseline NarxCare ORS and achievement of PASS threshold

After controlling for potential confounders, baseline NarxCare ORS of > 100 was associated with failure to achieve PASS thresholds for HOOS Pain, PS, and JR. For pain PASS failure, baseline ORS scores of 100–199 (OR: 1.45, 95% CI: 1.23–1.71, *p* < 0.001), 200–299 (OR: 1.57, 95% CI: 1.30–1.90, *p* < 0.001), 300–399 (OR: 2.03, 95% CI: 1.57–2.61, *p* < 0.001), 400–499 (OR: 2.85, 95% CI: 1.93–4.20, *p* < 0.001), and ≥ 500 (OR: 2.19, 95% CI: 1.31–3.66, *p* = 0.003) were significantly associated. For PS PASS failure, ORS scores of 100–199 (OR: 1.47, 95% CI: 1.22–1.75, *p* < 0.001), 200–299 (OR: 1.44, 95% CI: 1.17–1.77, *p* = 0.001), 300–399 (OR: 1.86, 95% CI: 1.37–2.37, *p* < 0.001), 400–499 (OR: 2.85, 95% CI: 1.74–4.04, *p* < 0.001), and ≥ 500 (OR: 3.09, 95% CI: 1.84–5.21, *p* < 0.001) showed significant associations. Regarding JR PASS failure, ORS scores of 100–199 (OR: 1.26, 95% CI: 1.08–1.49, *p* = 0.004), 200–299 (OR: 1.37, 95% CI: 1.14–1.64, *p* = 0.001), 300–399 (OR: 1.91, 95% CI: 1.48–2.46, *p* < 0.001), 400–499 (OR: 2.53, 95% CI: 1.67–3.83, *p* < 0.001), and ≥ 500 (OR: 2.67, 95% CI: 1.56–4.57, *p* < 0.001) were significantly associated (Table 2).

### Baseline NarxCare ORS and satisfaction at 1-year

Baseline ORS scores of 100–199 were associated with higher odds of dissatisfaction (OR: 1.58, 95% CI: 1.26–1.99, *p* < 0.001), as were scores of 200–299 (OR: 1.36, 95% CI: 1.04–1.77, *p* = 0.026), 300–399 (OR: 1.94, 95% CI: 1.39–2.71, *p* < 0.001), 400–499 (OR: 2.96, 95% CI: 1.86–4.71, *p* < 0.001), and ≥ 500 (OR: 2.43, 95% CI: 1.31–4.52, *p* = 0.005). These results indicate that increasing baseline ORS is strongly predictive of dissatisfaction at 1 year, with the highest odds of dissatisfaction observed among patients with ORS scores in the 400–499 range (Table 3).

## Discussion

The opioid crisis has profoundly influenced healthcare systems, with its implications for THA outcomes being extensively examined in contemporary research [3, 23]. However, the effects of nonopioid prescription medications, including sedatives and stimulants, remain underexplored, despite their significant prevalence of misuse [7]. While a NarxCare ORS exceeding 300 has been linked to increased healthcare utilization post-THA, its specific impact on PROMs remains inadequately characterized. Addressing this shortfall, the current cohort study of 5,424 patients undergoing THA demonstrates that a NarxCare ORS greater than 100 is associated with a failure to achieve meaningful improvements in patient-perceived hip pain and function, as well as satisfaction at one year postoperatively, with the odds of failure increasing as the score rises. These findings underscore the dose- and duration-dependent relationship between prescription drug use and PROMs following THA. Consequently, surgeons should leverage this readily available scoring system to inform patient counseling and facilitate multidisciplinary interventions aimed at mitigating the adverse effects of prescription drug use.

In the present study, 46.1% of patients had a preoperative NarxCare ORS of 0, indicating that they were prescription drug-naïve in terms of narcotics, sedatives, or stimulants. This aligns with previous literature examining prescription drug use prior to THA. Bedard et al. [24] reported that 38% of THA patients were opioid users preoperatively, while Rajamaki et al. [25] found that 12% of their cohort was on benzodiazepines prior to surgery. Since the NarxCare ORS combines narcotic, sedative, and stimulant usage into a single risk metric, our results are consistent with these studies when considering the individual components of the ORS. These findings reinforce that nearly half of the patients undergoing THA have a history of prescription drug use, underscoring the importance of studying its impact on patient-perceived outcomes.

The postoperative increase in median ORS observed in this study provides important insight into the evolving prescription-risk profile of patients after THA. Although preoperative ORS was evaluated as the primary predictor of 1-year PROMs and satisfaction, the rise in ORS after surgery suggests that ORS may also function as a dynamic perioperative marker rather than only a baseline risk factor. This increase may be partly attributable to expected postoperative analgesic prescribing for acute surgical pain; however, persistence of elevated scores through 1 year may also reflect prolonged medication requirements, persistent pain, continuation of preexisting narcotic, sedative, or stimulant prescriptions, prescription overlap, or fragmented prescribing across multiple providers. Clinically, these findings suggest that serial ORS assessment may have utility beyond preoperative counseling, potentially helping identify patients who require closer pain monitoring, medication reconciliation, coordinated prescribing, multimodal analgesia optimization, or referral to pain-management and primary-care teams. Furthermore, postoperative ORS trajectories may represent a useful marker of postoperative recovery complexity and healthcare utilization risk, particularly if persistently elevated scores are associated with delayed functional recovery, prolonged pharmacologic pain control, emergency department visits, or readmissions. Future research should evaluate whether longitudinal changes in ORS provide incremental prognostic value for PROM recovery, satisfaction, persistent pain, and healthcare utilization after THA.

Patients with higher preoperative ORS also presented with worse baseline patient-reported pain and function. Baseline HOOS Pain, HOOS-PS, and HOOS-JR scores progressively decreased across increasing ORS categories, indicating that patients with greater preoperative prescription burden entered THA with a worse symptom and functional profile. This finding provides important context for interpreting postoperative outcomes, as patients with elevated ORS may represent a more clinically complex subgroup with greater baseline pain burden and potentially more challenging perioperative pain-management needs. Importantly, the association between ORS and 1-year outcomes persisted despite adjustment for baseline PROM phenotype, which incorporates baseline pain, physical function, and mental health status. Thus, elevated ORS may capture additional clinical and prescription-related complexity beyond baseline symptom severity alone.

Our findings indicate that the achievement of MCID was more attainable for patients across all levels of prescription drug use. However, patients with baseline NarxCare ORS scores ≥ 100 demonstrated higher odds of failing to achieve PASS for HOOS Pain, HOOS-PS, and HOOS-JR. Additionally, the risk of failing to reach an acceptable symptomatic state for hip pain and function increased significantly with rising prescription drug use, suggesting a dose-dependent relationship. For example, patients in the 100–199 ORS group had a 45% increased risk of failing to achieve PASS thresholds for HOOS pain, while those in the 400–499 ORS group experienced a 185% increased risk. This difference highlights that MCID is based on relative improvement from baseline, whereas PASS thresholds represent an absolute symptomatic target that must be reached at one year postoperatively. Although this is the first to explore the association between NarxCare ORS and PROMs, these results are consistent with prior studies investigating the relationship between preoperative opioid use and patient-reported outcomes. Bonner et al. [11] found that while all patients experienced improvements after THA, preoperative opioid users had significantly lower PROMs and longer hospital stays. Similarly, Singh et al. [26] demonstrated that preoperative opioid users exhibited significantly lower baseline PROMs but did not differ significantly from non-users postoperatively, suggesting a larger delta improvement but similar final outcomes. In contrast, Huang et al. [27] reported that preoperative opioid use in an Australian cohort was not associated with inferior outcomes or satisfaction, potentially reflecting differences in healthcare systems, patient populations, or perioperative management. These results underscore the need for further research into preoperative optimization strategies for patients with higher prescription drug use, beyond just focusing on opioids, to enhance postoperative patient perceived outcomes and improve their likelihood of reaching acceptable symptomatic states.

Emara et al. [12] previously demonstrated that a NarxCare ORS of 300 or greater was significantly associated with longer hospital stays, higher 90-day readmission rates, and non-home discharge dispositions. In contrast, the current study found that patients in ORS categories beginning at ≥ 100 had lower likelihood of achieving satisfaction and PASS for hip pain and function at 1 year postoperatively. More recently, Emara et al. demonstrated that preoperative NarxCare ORS greater than 200 was associated with worse 1-year PROMs and dissatisfaction after primary total knee arthroplasty [28]. The present study extends these findings to primary THA, demonstrating that elevated preoperative ORS is similarly associated with lower likelihood of achieving clinically meaningful improvement, PASS, and satisfaction at 1 year. This reinforces the importance of using NarxCare ORS as a readily available tool to assess preoperative prescription drug use patterns, encompassing narcotics, sedatives, and stimulants, to guide patient-centered discussions. By incorporating this metric into preoperative evaluations, surgeons can counsel patients on how their baseline prescription drug use may impact their postoperative recovery and expected improvements, enabling more personalized care planning and earlier involvement of multidisciplinary approach to lower the impact of prescription drug use on THA outcomes.

The findings of this study should be viewed in the context of its limitations. First, the NarxCare ORS has a lag between a patient’s use of prescription drugs and the score being updated in the system. The frequency of updates may depend on the integration between the PDMP, NarxCare platform, and the hospital’s EMR system, as well as local regulations. This means the score at the time of evaluation may not always reflect the most current prescription drug use. Second, the maximum follow-up within this study was limited to 1 year; therefore, no conclusions surrounding longer term outcomes may be made at this time. However, it has been suggested that clinically meaningful PROMs score do not change drastically after 1-year [29–32]. Additionally, the MCID analyses may be limited by the high overall rate of MCID achievement, which reduced the number of MCID failure events within individual ORS categories and may have limited statistical power for some category-specific comparisons. Therefore, non-significant MCID findings should be interpreted cautiously in the context of the more consistent associations observed for PASS achievement and satisfaction. Moreover, while ORS categories were associated with 1-year PROMs and satisfaction, the present study did not derive or validate a data-driven ORS cutoff for clinical decision-making. Future studies should perform ROC-based analyses with external validation to determine whether specific ORS cutoffs can reliably identify patients at increased risk for worse patient-reported recovery after THA. Further, this study did not include clinician-administered measures such as the Harris Hip Score, which is a widely validated outcome measure after THA, as it was not routinely and consistently collected in the institutional registry during the study period and therefore could not be incorporated. Instead, this analysis focused on HOOS subdomains, which are validated hip-specific PROMs, and align with the study objective of evaluating patient-perceived pain, function, clinically meaningful improvement, and satisfaction after THA. Finally, this study was conducted at a single academic health network, which may limit the generalizability of the results to other institutions or healthcare settings. However, the analysis accounted for socioeconomic status, and the large cohort size strengthens the reliability of the findings.

**Table 1:** Multivariable logistic regression model, using failure to reach MCID improvements in HOOS Pain, PS, and JR as outcomes

Predictors	Pain MCID (failure)		PS MCID (failure)		JR MCID (failure)	
	OR (95%CI)	*P*-value	OR (95%CI)	*P*-value	OR (95%CI)	*P*-value
Baseline ORS (1–99 v 0)	1.47 (0.57–3.80)	0.426	0.84 (0.42–1.67)	0.621	1.93 (0.95–3.91)	0.068
Baseline ORS (100–199 v 0)	1.91 (1.32–2.77)	**0.001**	1.17 (0.91–1.50)	0.212	1.54 (1.10–2.15)	**0.012**
Baseline ORS (200–299 v 0)	1.53 (0.98–2.41)	0.063	1.32 (0.98–1.76)	0.065	1.56 (1.06–2.30)	**0.024**
Baseline ORS (300–399 v 0)	1.56 (0.82–2.97)	0.173	1.25 (0.81–1.91)	0.313	1.80 (1.06–3.06)	**0.029**
Baseline ORS (400–499 v 0)	2.12 (0.81–5.54)	0.124	2.17 (1.16–4.05)	**0.016**	4.36 (2.22–8.56)	**< 0.001**
Baseline ORS ( > = 500 v 0)	4.33 (1.70–11.03)	**0.002**	5.23 (2.71–10.07)	**< 0.001**	3.71 (1.61–8.57)	**0.002**

This model was adjusted for Age, Sex, BMI, Race, Education, Smoking, Area Deprivation Index, Charlson Comorbidity Index, Insurance, Diagnosis, Anesthesia, PROM Phenotype. Bold shows statistically significant results.

**Table 2:** Multivariable logistic regression model, using failure to reach PASS Thresholds in HOOS Pain, PS, and JR as outcomes

Predictors	Pain PASS (failure)		PS PASS (failure)		JR PASS (failure)	
	Estimate [95%CI]	*P*-value	Estimate [95%CI]	*P*-value	Estimate [95%CI]	*P*-value
Baseline ORS (1–99 v 0)	1.37 (0.93–2.03)	0.113	1.46 (0.96–2.22)	0.079	1.35 (0.93–1.97)	0.116
Baseline ORS (100–199 v 0)	1.45 (1.23–1.71)	**< 0.001**	1.47 (1.22–1.75)	**< 0.001**	1.26 (1.08–1.49)	**0.004**
Baseline ORS (200–299 v 0)	1.57 (1.30–1.90)	**< 0.001**	1.44 (1.17–1.77)	**0.001**	1.37 (1.14–1.64)	**0.001**
Baseline ORS (300–399 v 0)	2.03 (1.57–2.61)	**< 0.001**	1.80 (1.37–2.37)	**< 0.001**	1.91 (1.48–2.46)	**< 0.001**
Baseline ORS (400–499 v 0)	2.85 (1.93–4.20)	**< 0.001**	2.65 (1.74–4.04)	**< 0.001**	2.53 (1.67–3.83)	**< 0.001**
Baseline ORS ( > = 500 v 0)	2.19 (1.31–3.66)	**0.003**	3.09 (1.84–5.21)	**< 0.001**	2.67 (1.56–4.57)	**< 0.001**

This model was adjusted for Age, Sex, BMI, Race, Education, Smoking, Area Deprivation Index, Charlson Comorbidity Index, Insurance, Diagnosis, Anesthesia, PROM Phenotype. Bold shows statistically significant results.

**Table 3:** Multivariable logistic regression model, using dissatisfaction at 1-Year as the outcome

Predictors	Satisfaction (No)	
	Estimate [95%CI]	*P*-value
Baseline ORS (1–99 v 0)	1.36 (0.79–2.36)	0.272
Baseline ORS (100–199 v 0)	1.58 (1.26–1.99)	**< 0.001**
Baseline ORS (200–299 v 0)	1.36 (1.04–1.77)	**0.026**
Baseline ORS (300–399 v 0)	1.94 (1.39–2.71)	**< 0.001**
Baseline ORS (400–499 v 0)	2.96 (1.86–4.71)	**< 0.001**
Baseline ORS ( > = 500 v 0)	2.43 (1.31–4.52)	**0.005**

This model was adjusted for Age, Sex, BMI, Race, Education, Smoking, Area Deprivation Index, Charlson Comorbidity Index, Insurance, Diagnosis, Anesthesia, PROM Phenotype. Bold shows statistically significant results.

## Conclusion

A NarxCare ORS of ≥ 100 reduces the likelihood of achieving clinically meaningful improvements in hip pain, function, and patient satisfaction at one year postoperatively, with the odds of failure increasing as the score increases. Surgeons can therefore leverage this readily available numerical tool to identify patients with increased prescription drug use patterns and facilitate patient-centered discussions, setting realistic expectations about postoperative outcomes and exploring strategies to reduce prescription drug use before their THA. A multidisciplinary care approach may be warranted to address the potential adverse impacts of sedative, opioid, or stimulant drug use on patient perceived outcomes at 1-year postoperatively.

## Supplementary Information

Below is the link to the electronic supplementary material.

Supplementary Material 1

## Data Availability

No datasets were generated or analysed during the current study.
