# Supplementary material for: Preoperative NarxCare overdose risk scores greater than 100 are associated with worse PROMs improvements and dissatisfaction after primary THA
Source: Arch Orthop Trauma Surg. 2026 Jun 18;146(1):223. doi: 10.1007/s00402-026-06372-7 (PMC13279391; doi:10.1007/s00402-026-06372-7)
Supplement: Supplementary file 1 — Supplementary Material 1 [file 402_2026_6372_MOESM1_ESM.docx]

| **Appendix Table 1. Patient Characteristics by Baseline NarxCare Overdose Risk Score** | | | | | | | | | | |
| --- | --- | --- | --- | --- | --- | --- | --- | --- | --- | --- |
| **Variable** | **Level** | **All (n=5424)** | **0 (n=2501)** | **1-99 (n=151)** | **100-199 (n=1336)** | **200-299 (n=876)** | **300-399 (n=357)** | **400-499 (n=131)** | **>500 (n=72)** | **P-value** |
| **Age** |  | 67.0 [60.0;73.0] | 67.0 [60.0;73.0] | 67.0 [61.0;73.0] | 68.0 [61.0;74.0] | 66.0 [60.0;73.0] | 67.0 [60.0;71.0] | 64.0 [57.5;71.5] | 60.0 [53.8;67.0] | **<0.001** |
| **Sex** | F | 3134 (57.8%) | 1388 (55.5%) | 98 (64.9%) | 808 (60.5%) | 505 (57.6%) | 217 (60.8%) | 74 (56.5%) | 44 (61.1%) | **0.027** |
|  | M | 2290 (42.2%) | 1113 (44.5%) | 53 (35.1%) | 528 (39.5%) | 371 (42.4%) | 140 (39.2%) | 57 (43.5%) | 28 (38.9%) |  |
| **BMI** |  | 29.2 [25.7;33.7] | 28.7 [25.2;33.0] | 29.5 [25.7;34.0] | 29.5 [26.0;34.0] | 29.8 [26.0;34.3] | 30.1 [26.1;34.7] | 30.7 [26.8;35.2] | 30.4 [26.1;35.7] | **<0.001** |
| **Education** |  | 14.0 [12.0;16.0] | 15.0 [12.0;16.0] | 14.0 [12.0;16.0] | 14.0 [12.0;16.0] | 14.0 [12.0;16.0] | 14.0 [12.0;16.0] | 14.0 [12.0;16.0] | 14.0 [12.0;16.0] | **<0.001** |
| **ADI** |  | 52.0 [31.0;71.0] | 49.0 [30.0;69.0] | 56.0 [36.5;73.0] | 53.0 [32.0;75.0] | 53.0 [33.0;70.8] | 53.0 [31.0;73.0] | 53.0 [36.0;70.0] | 60.0 [35.0;80.5] | **<0.001** |
| **Race** | White | 4788 (89.4%) | 2222 (89.8%) | 130 (87.8%) | 1164 (88.4%) | 767 (88.9%) | 325 (91.0%) | 117 (92.9%) | 63 (87.5%) | 0.518 |
|  | Non-white | 568 (10.6%) | 252 (10.2%) | 18 (12.2%) | 152 (11.6%) | 96 (11.1%) | 32 (8.96%) | 9 (7.14%) | 9 (12.5%) |  |
| **Smoking** | Never | 2985 (55.0%) | 1500 (60.0%) | 72 (47.7%) | 707 (52.9%) | 446 (50.9%) | 177 (49.6%) | 55 (42.0%) | 28 (38.9%) | **<0.001** |
|  | Quit 6m+ | 1831 (33.8%) | 773 (30.9%) | 60 (39.7%) | 483 (36.2%) | 305 (34.8%) | 130 (36.4%) | 54 (41.2%) | 26 (36.1%) |  |
|  | Quit 0-6m | 192 (3.54%) | 75 (3.00%) | 4 (2.65%) | 52 (3.89%) | 32 (3.65%) | 15 (4.20%) | 10 (7.63%) | 4 (5.56%) |  |
|  | Current | 416 (7.67%) | 153 (6.12%) | 15 (9.93%) | 94 (7.04%) | 93 (10.6%) | 35 (9.80%) | 12 (9.16%) | 14 (19.4%) |  |
| **CCI** |  | 0.00 [0.00;2.00] | 0.00 [0.00;1.00] | 1.00 [0.00;2.00] | 1.00 [0.00;2.00] | 1.00 [0.00;2.00] | 1.00 [0.00;2.00] | 1.00 [0.00;2.00] | 1.00 [0.00;3.00] | **<0.001** |
| **Categorical CCI** | 0 | 2808 (52.7%) | 1465 (60.0%) | 67 (45.3%) | 619 (46.9%) | 404 (47.1%) | 171 (48.2%) | 52 (40.6%) | 30 (41.7%) | **<0.001** |
|  | 1 | 1063 (20.0%) | 449 (18.4%) | 29 (19.6%) | 293 (22.2%) | 181 (21.1%) | 74 (20.8%) | 27 (21.1%) | 10 (13.9%) |  |
|  | 2 | 711 (13.4%) | 284 (11.6%) | 25 (16.9%) | 192 (14.5%) | 128 (14.9%) | 46 (13.0%) | 25 (19.5%) | 11 (15.3%) |  |
|  | 3+ | 743 (14.0%) | 245 (10.0%) | 27 (18.2%) | 217 (16.4%) | 145 (16.9%) | 64 (18.0%) | 24 (18.8%) | 21 (29.2%) |  |
| **Insurance** | Medicaid/Medicare | 3297 (60.9%) | 1421 (56.9%) | 103 (68.2%) | 859 (64.3%) | 543 (62.0%) | 241 (67.9%) | 81 (61.8%) | 49 (69.0%) | **<0.001** |
|  | Commercial/Self-pay | 2118 (39.1%) | 1075 (43.1%) | 48 (31.8%) | 476 (35.7%) | 333 (38.0%) | 114 (32.1%) | 50 (38.2%) | 22 (31.0%) |  |
| **Diagnosis** | Non-OA | 528 (9.73%) | 199 (7.96%) | 13 (8.61%) | 106 (7.93%) | 111 (12.7%) | 50 (14.0%) | 26 (19.8%) | 23 (31.9%) | **<0.001** |
|  | OA | 4896 (90.3%) | 2302 (92.0%) | 138 (91.4%) | 1230 (92.1%) | 765 (87.3%) | 307 (86.0%) | 105 (80.2%) | 49 (68.1%) |  |
| **Anesthesia** | General | 1722 (31.8%) | 741 (29.7%) | 53 (35.1%) | 413 (30.9%) | 297 (33.9%) | 131 (36.9%) | 58 (44.3%) | 29 (40.8%) | **0.002** |
|  | Spinal | 3494 (64.5%) | 1662 (66.6%) | 90 (59.6%) | 879 (65.8%) | 550 (62.8%) | 205 (57.7%) | 69 (52.7%) | 39 (54.9%) |  |
|  | Other | 198 (3.66%) | 92 (3.69%) | 8 (5.30%) | 43 (3.22%) | 29 (3.31%) | 19 (5.35%) | 4 (3.05%) | 3 (4.23%) |  |
| **PROM Phenotype** | P-PS-MCS- | 1268 (23.4%) | 421 (16.8%) | 42 (27.8%) | 317 (23.7%) | 278 (31.7%) | 120 (33.6%) | 52 (39.7%) | 38 (52.8%) | **<0.001** |
|  | P+PS+MCS+ | 1452 (26.8%) | 810 (32.4%) | 26 (17.2%) | 350 (26.2%) | 182 (20.8%) | 69 (19.3%) | 10 (7.63%) | 5 (6.94%) |  |
|  | P+PS+MCS- | 831 (15.3%) | 406 (16.2%) | 28 (18.5%) | 194 (14.5%) | 134 (15.3%) | 45 (12.6%) | 19 (14.5%) | 5 (6.94%) |  |
|  | P+PS-MCS+ | 278 (5.13%) | 152 (6.08%) | 4 (2.65%) | 65 (4.87%) | 33 (3.77%) | 21 (5.88%) | 2 (1.53%) | 1 (1.39%) |  |
|  | P+PS-MCS- | 278 (5.13%) | 130 (5.20%) | 12 (7.95%) | 71 (5.31%) | 36 (4.11%) | 16 (4.48%) | 9 (6.87%) | 4 (5.56%) |  |
|  | P-PS+MCS+ | 302 (5.57%) | 145 (5.80%) | 5 (3.31%) | 96 (7.19%) | 33 (3.77%) | 15 (4.20%) | 4 (3.05%) | 4 (5.56%) |  |
|  | P-PS+MCS- | 338 (6.23%) | 129 (5.16%) | 18 (11.9%) | 86 (6.44%) | 60 (6.85%) | 25 (7.00%) | 13 (9.92%) | 7 (9.72%) |  |
|  | P-PS-MCS+ | 677 (12.5%) | 308 (12.3%) | 16 (10.6%) | 157 (11.8%) | 120 (13.7%) | 46 (12.9%) | 22 (16.8%) | 8 (11.1%) |  |

Continuous variables presented as Median [IQR]. Categorical variables presented as N (column %).

| **Appendix Table 2. Outcomes by Baseline NarxCare Overdose Risk Score** | | | | | | | | | | | |
| --- | --- | --- | --- | --- | --- | --- | --- | --- | --- | --- | --- |
| **Variable** | **Level** | **All (n=5414)** | **0 (n=2496)** | **1-99 (n=151)** | **100-199 (n=1335)** | **200-299 (n=875)** | **300-399 (n=355)** | **400-499 (n=131)** | **>500 (n=72)** | **P-value** | **N** |
| **Baseline HOOS-Pain** |  | 37.5 [25.0;47.5] | 40.0 [30.0;50.0] | 35.0 [25.0;45.0] | 37.5 [27.5;47.5] | 35.0 [22.5;42.5] | 32.5 [20.0;42.5] | 27.5 [17.5;37.5] | 22.5 [7.50;32.5] | **<0.001** | 5424 |
| **Baseline HOOS-PS** |  | 53.9 [44.1;66.1] | 53.9 [44.1;66.1] | 53.9 [38.4;62.3] | 53.9 [44.1;66.1] | 49.2 [38.4;62.3] | 49.2 [38.4;62.3] | 44.1 [32.1;58.3] | 38.4 [17.6;53.9] | **<0.001** | 5424 |
| **Baseline HOOS-JR** |  | 43.3 [36.4;53.0] | 46.7 [36.4;56.0] | 43.3 [32.7;53.0] | 43.3 [36.4;53.0] | 39.9 [29.0;49.9] | 39.9 [29.0;49.9] | 36.4 [25.1;46.7] | 34.5 [19.5;43.3] | **<0.001** | 5424 |
| **1-Year HOOS-Pain** |  | 95.0 [80.0;100] | 97.2 [85.0;100] | 92.5 [78.1;100] | 92.5 [77.5;100] | 92.5 [77.5;100] | 90.0 [72.5;97.5] | 85.0 [65.0;99.4] | 82.5 [57.5;98.1] | **<0.001** | 5387 |
| **1-Year HOOS-PS** |  | 95.4 [83.6;100] | 95.4 [87.3;100] | 91.2 [80.0;100] | 95.4 [80.0;100] | 91.2 [80.0;100] | 91.2 [76.6;100] | 91.2 [69.6;100] | 83.6 [62.3;97.7] | **<0.001** | 5085 |
| **1-Year HOOS-JR** |  | 85.3 [73.5;100] | 92.3 [76.8;100] | 85.3 [70.4;100] | 85.3 [73.5;100] | 85.3 [70.4;100] | 80.6 [67.5;92.3] | 75.1 [64.7;100] | 73.5 [58.9;88.8] | **<0.001** | 4934 |
| **PASS** |  | 4755 (88.9%) | 2268 (92.0%) | 129 (87.8%) | 1151 (87.1%) | 756 (87.7%) | 296 (83.6%) | 100 (77.5%) | 55 (77.5%) | **<0.001** | 5351 |
| **Pain MCID (8.35)** | Achieved | 5195 (96.4%) | 2426 (97.4%) | 145 (96.7%) | 1258 (95.1%) | 835 (96.3%) | 340 (95.8%) | 125 (96.2%) | 66 (91.7%) | **0.005** | 5387 |
| **PS MCID (9.47)** | Achieved | 4588 (90.2%) | 2158 (91.2%) | 125 (89.9%) | 1123 (89.7%) | 729 (90.0%) | 301 (90.7%) | 99 (86.8%) | 53 (74.6%) | **0.001** | 5085 |
| **JR MCID (7.76)** | Achieved | 4672 (94.7%) | 2204 (96.1%) | 131 (92.3%) | 1131 (93.8%) | 745 (94.3%) | 301 (93.8%) | 102 (89.5%) | 58 (86.6%) | **<0.001** | 4934 |
| **PASS Threshold Pain (80.6)** | Achieved | 3986 (74.0%) | 2009 (80.7%) | 106 (70.7%) | 943 (71.3%) | 595 (68.6%) | 224 (63.1%) | 70 (53.8%) | 39 (54.2%) | **<0.001** | 5387 |
| **PASS Threshold PS (83.6)** | Achieved | 3951 (77.7%) | 1981 (83.7%) | 101 (72.7%) | 938 (74.9%) | 596 (73.6%) | 229 (69.0%) | 70 (61.4%) | 36 (50.7%) | **<0.001** | 5085 |
| **PASS Threshold JR (76.8)** | Achieved | 3288 (66.6%) | 1679 (73.2%) | 85 (59.9%) | 781 (64.8%) | 492 (62.3%) | 172 (53.6%) | 52 (45.6%) | 27 (40.3%) | **<0.001** | 4934 |
| Continuous variables presented as Median (IQR). Categorical variables presented as N (column %). | | | | | | | | | | | |
